# Analysis of hemorrhage upon ultrasound-guided percutaneous renal biopsy in China: a retrospective study

**DOI:** 10.1007/s11255-023-03860-2

**Published:** 2023-11-22

**Authors:** Fang-fang Li, Yu-xia Guan, Tong-xin Li, Di Jiang, Zi-xia He, Peng Xia, Xue-song Zhao

**Affiliations:** 1 Department of Endocrinology, Peking Union Medical College Hospital, Peking, China; 2 Department of Nephrology, Peking Union Medical College Hospital, No.1 Shuaifuyuan Wangfujing Dongcheng District, Beijing, 100730 China; 3 Intensive Care Unit, Peking Union Medical College Hospital, Peking, China

**Keywords:** Chronic kidney disease, Percutaneous renal biopsy, Post-biopsy bleeding complication, Prevalence, Associated factor

## Abstract

**Purpose:**

Ultrasound-guided percutaneous renal biopsy (PRB) has been considered as a golden standard for CKD diagnosis and is employed to identify potential therapeutic targets since 1950s. Post-biopsy hemorrhage is the most common complication, while severe bleeding complication might cause nephrectomy or death. Therefore, how to reduce the occurrence of complications while ensuring the success of PRB is always a clinical research topic.

**Methods:**

This study retrospectively collected and established a renal biopsy database of each patient who underwent ultrasound-guided PRB at a tertiary teaching hospital from September 2017 to December 2020 through the Health Information System. All the data were statistically processed by SPSS software.

**Results:**

A total of 1146 patients underwent PRB for various reasons. The overall rate of post-biopsy hemorrhage was 37.70% (432/1146). Of those bleedings, minor bleeding after PRB was found in 337 (29.41%), middle bleeding 84 (7.33%), major bleeding 11 (0.96%). Besides that, there were 96 patients (8.38%) reported their discomfort symptoms. There was no death. Females were at significantly increased risk of hemorrhagic complication than males (OR = 2.017, CI = 1.531–2.658). While the risk for hemorrhagic complication significantly decreased as BMI and platelet before renal biopsy increased (OR = 0.956, CI = 0.924–0.989; OR = 0.998, CI = 0.996–1.000). As the APTT time prolonged, the risk for hemorrhagic complication significantly increased (OR = 1.072, CI = 1.023–1.123). Those patients whose albumin were higher, also had higher risk for hemorrhagic complication than other patients (OR = 1.020, CI = 1.000–1.041). Specifically, postoperative urination within 4 h increased the risk for hemorrhagic complication (OR = 1.741, CI = 1.176–2.576).

**Conclusion:**

Our analysis finds that the incidence of post-biopsy bleeding complication is 37.70%, and its risk is associated with female, lower BMI, lower platelet before renal biopsy, prolonged APTT, higher albumin, and postoperative urination within 4 h. The findings highlighted the importance of perioperative management for renal biopsy, including adequate risk assessment, tailored careful observation after PRB. And medical staff should pay more attention to fluid management after ultrasound-guided PRB.

## Introduction

Chronic kidney disease (CKD) is defined as abnormalities of kidney structure or function, present for more than 3 months, with implications for health and CKD is classified based on cause, GFR category, and albuminuria category (CGA) [1]. CKD is worldwide public health problem affecting millions of people and has rapidly increased in prevalence due in part to the aging population increasing with hypertension and diabetes in recent years. The global estimated prevalence of CKD is 13.4% [2]. In addition, according to a nationwide survey conducted between 2009 and 2010 by Zhang et al. [3], the overall prevalence of CKD in China is 10.8%. Over the past 10 years, although there is no update nationwide data, the incidence of CKD is still believed be on the rising trend attributed to the increasing amount of diabetic or hypertensive populations. The burden of CKD is not restricted to its effect on demands for renal replacement therapy; the disease has other major effects, including mortality and cardiovascular events. Both the developing and developed countries face the challenge of an increasing burden of CKD [4].

Identifying risk factors and corresponding health management measures for high-risk individuals can decrease the progression of CKD, as well as to reduce its complications, death, and the disease burden. However, just relying on clinical presentation or laboratory examination alone are not enough to well guide the diagnosis, treatment and prognosis. Ultrasound-guided percutaneous renal biopsy (PRB) has been considered as a golden standard for CKD diagnosis and is employed to identify potential therapeutic targets since 1950s. Ultrasound-guided PRB is recommended in patients with significant proteinuria, hematuria, acute kidney injury, unexpected worsening of renal function, and allograft dysfunction after excluding pre- and post-renal causes [5]. The advent of improved imaging techniques, ultrasound equipment, automated spring-loaded biopsy devices, and biopsy needles over the years has increased the general safety of the procedure and the ability to obtain adequate renal tissue for diagnosis. However, as an invasive procedure, there still occurs procedure-related complications, which have negative impact on patient safety, prolong their hospitalization time and increase their burden.

Post-biopsy hemorrhage is the most common complication, while severe bleeding complication might cause nephrectomy or death. According to other study, 89% of hemorrhagic complications have been reported to occur within 24 h [6]. How to reduce the occurrence of complications while ensuring the success of PRB is always a clinical research topic. The related risk factors to hemorrhagic complication can be classified into four categories: (1) patient factors: age, gender, platelet count, hemoglobin concentration, kidney size, body mass index (BMI), coagulation status, and period adherence; (2) disease factors: severity of kidney dysfunction, clinicopathological diagnosis, comorbidities, such as hypertension; (3) operational factors: number of passes with the biopsy needle, and size of side slot cutting needle; (4) drug-related factors: taking antiplatelet aggregation drugs before PRB, prophylactic use of hemostatic drugs. Above all the risk factors, on the one hand, there remains interest in potentially modifiable risk factors, such as hypertension. On the other hand, there might be other risk factors that are still not found. Therefore, we not only collected these variables to identify the risk factors for our patients, but also increased other variables, such as 24-h intake and urine volume after puncture, and whether urinating within 4 h after PRB. Given the importance of PRB and risk reduction prior to post-biopsy bleeding, here we sought to determine the rate of bleeding complications after PRB and explore its related factors.

## Methods

### Data collection

This study retrospectively collected and established a renal biopsy database of each patient who underwent ultrasound-guided PRB at a tertiary teaching hospital from September 2017 to December 2020 through the Health Information System. The variables collected in this study include: demographic characteristics (patient’s age, gender, educational level, weight and height), comorbidities (hypertension, diabetes, and immune diseases), type of kidney (native or allograft), vital signs (brachial artery systolic pressure and diastolic pressure), laboratory results before PRB (albumin, hemoglobin concentration, platelet count, serum creatinine value, prothrombin time, activated partial thromboplastin time, and international normalized ratio), disease duration, size of renal, cortical thickness, the number of biopsy needle pass, 24-h intake and urine volume after puncture and urination time after PRB. A standard data extraction template was used to collect this detailed information.

### Programed PRB operation procedure

Nephrologists, who had undergone unified training and passed examination, performed the biopsies with ultrasound experts assisted localization. BARD automatic biopsy gun and matching 16-gauge or 18-gauge biopsy needle was used to perform PRB. The renal cortex at the lateral edge of the lower pole of the left kidney was routinely selected as the sampling point for puncture. After routine skin disinfection and drape, 1% lidocaine was  used to anesthetize to the renal capsule layer by layer, and a 0.3 cm incision was made in the skin at the puncture point with a sharp scalpel. The nephrologists would activate the biopsy gun under real-time ultrasound guidance, and immediately pull out the puncture needle after cutting the material, and the assistant compressed the biopsy site for 10–15 min to stop bleeding. The technicians will check the samples immediately at the bedside. If the samples were unsatisfactory, the nephrologists would repeat the puncture.

### Periprocedural management of patients

Patients received periprocedural management, including: (1) preoperative education: All the patients were asked to stop using antiplatelet aggregation drugs at least 1 day [aspirin (> 7 days), dipyridamole (> 1 day), ticlopidine (> 5 days), clopidogrel (> 7 days), and anticoagulant medication at least 3 days [warfarin (> 3 days), non-vitamin K oral anticoagulants (> 1 day)] before their PRB. Besides that, the nephrologists would do pre-procedure assessments to minimize the risk of bleeding, including the blood routine and blood coagulation function test and blood pressure measurement of the patients. The nurses would conduct preoperative education to these patients, including informing them of the necessity of puncture, brief operation procedures, coordination during puncture, and postoperative precautions. The patients were also trained breath-holding coordination, defecation in bed, etc. (2) Intraoperative cooperation: patients were generally placed in the prone position, with a pillow under their abdomen. When the needle reached the renal capsule, the patient was instructed to hold his breath immediately. After the operation, the patient would be transported to the ward by a transfer bed to reduce unnecessary movement. (3) Postoperative observation: the nurses conducted 24-h careful observation for the patients after PRB to assure their safety. Post-procedural routine care involves vital signs monitoring (temperature, respiration, pulse, saturation and blood pressure), clinical presentation (urine color, time of urination, and 24-h intake and urine volume), and patient complaints (nausea, vomiting, and pain). The patient should be supine for 12 h and then remain at bedrest over 24 h. After 24 h, they could move on the ground under the nurse guidance. Perinephric hemorrhage was reviewed 48 h after surgery.

### Definition

Post-renal biopsy bleeding complications are classified as major, middle or minor. Defined minor bleeding complications as subcapsular perinephric hematoma (diameter < 5 cm), which could spontaneously be resolved without need for further clinical intervention. Middle bleeding complications were that include gross hematuria and/or subcapsular perinephric hematoma (diameter > 5 cm), which needed to prolong time in bed. Major bleeding complications were that hemorrhage, which requiring transfusion, bleeding with necessity of nephrectomy and death.

### Indications

The ultrasound-guided PRB was performed for certain patients with acute kidney injury (AKI), significant proteinuria, or hematuria, and unexpected worsening of renal function [5–8].

### Statistical analysis

All the data were statistically processed by SPSS software (version 19.0; SPSS, Chicago, IL, USA), the measurement data were expressed as mean ± standard deviation, and the counting data used were number (*n*) and percentage (%). Student’s *t* tests were used to compare between-group continuous variables, while Chi-square test was carried out to make between-group comparisons with categorical variables. *P* values < 0.05, in two-tailed tests, were considered statistically significant. Multivariable logistic regressions were used to analyze the hierarchical impact factors for post-biopsy hemorrhage.

## Results

A total of 1146 patients underwent PRB for various reasons*.* Table 1 shows that the three common reasons for patients who underwent renal biopsy were nephrotic syndrome (271), acute kidney injury (202), and worsening of renal function (189). Of those 1146 patients, there were 602 males (52.53%) and 544 females (47.47%). The average age of the patients was 42.78 ± 15.35. Most of them (99.13%) were native kidney. Nearly, 70% of the patients’ disease duration last more than 3 months. In addition, renal function as measured by the estimated glomerular filtration rate (eGFR) showed that the largest stage of CKD was stage 5 with 34.82% of cases, followed by stage 3 (26.88%), stage 4 (21.81%), stage 2 (10.73%) and stage 1 (5.76%). Out of all variables in this study, there were significant difference with age, gender, educational level, BMI, size of biopsied kidney, history of taking antiplatelet or anticoagulation, comorbidities (including hypertension, diabetes, and immune diseases), related library results before biopsy (including PLT, ALB, INR, PT, and APTT), pathologic diagnosis (including IgAN, MN, LN, DKD, and other diseases), and whether urinating within 4 h after puncture. The patient characteristics and univariate analysis results of this study are shown in Table 2.

**Table 1:** Common reasons for performing renal biopsy

Reasons	Number of patients
Nephrotic syndrome	271
Acute kidney injury	202
Worsening of renal function	189
Proteinuria/hematuria	157/4
Proteinuria with hematuria	97
Other reasons	226

Note: Other reasons included systemic lupus erythematosus, diabetes, and hypertension

**Table 2:** Univariate analysis of patient sociodemographic and clinical characteristics related to hemorrhagic complication

Variables	Total (*N* = 1146) *N* (%) or median	With bleeding complication *N* (%) or median	No bleeding complication *N* (%) or median	t/χ^2^/Z	*P* value
Age (years)	42.78 ± 15.35	40.45 ± 15.03	44.18 ± 15.38	4.013	< 0.001
Gender (%)				48.293	< 0.001
Male	602 (52.53)	170 (28.24)	432 (71.76)		
Female	544 (47.47)	262 (48.16)	282 (51.84)		
Educational level				− 2.753	0.006
Primary school or below	105 (9.16)	35 (33.33)	70 (66.67)		
Associate degree	524 (45.72)	179 (34.16)	345 (65.84)		
Bachelor degree or above	517 (45.11)	218 (42.17)	299 (57.83)		
BMI (kg/m^2^)	25.20 ± 4.22	24.46 ± 4.18	25.65 ± 4.18	4.688	< 0.001
Native kidney				0.692	0.405
Yes	1136 (99.13)	430 (37.85)	706 (62.15)		
No	10 (0.87)	2 (20.00)	8 (80.00)		
Length of biopsied kidney (cm)	10.92 ± 1.07	10.77 ± 1.07	11.00 ± 1.06	3.538	< 0.001
Cortical thickness (cm)	0.74 ± 0.20	0.73 ± 0.21	0.74 ± 0.19	0.459	0.646
Disease duration				0.816	0.366
< 3 months	348 (30.37)	138 (39.66)	210 (60.34)		
≥ 3 months	798 (69.63)	294 (36.84)	504 (63.16)		
History of taking antiplatelet or anticoagulation				8.004	0.005
Yes	123 (10.73)	32 (26.02)	91 (79.98)		
No	1023 (89.27)	400 (39.10)	623 (60.90)		
History of immune diseases				9.278	0.002
Yes	135 (11.78)	67 (49.63)	68 (50.37)		
No	1011 (88.22)	365 (36.10)	646 (63.90		
History of diabetes				5.090	0.024
Yes	176 (15.36)	53 (30.11)	123 (69.89)		
No	970 (84.64)	379 (39.07)	591 (60.93)		
History of hypertension				5.101	0.024
Yes	643 (56.11)	224 (34.84)	419 (65.16)		
No	503 (43.89)	208 (41.35	295 (58.65)		
SBP before biopsy (mmHg)				− 0.165	0.869
< 90	3 (0.26)	1 (33.33)	2 (66.67)		
90–140	949 (82.81)	359 (37.83)	590 (62.17)		
≥ 140	194 (16.93)	72 (37.11)	122 (62.89)		
DBP before biopsy (mmHg)				− 0.228	0.819
< 60	65 (5.67)	28 (43.08)	37 (56.92)		
60–90	910 (79.41)	338 (37.14)	572 (62.86)		
≥ 90	171 (14.92)	66 (38.60)	105 (61.40)		
PLT before biopsy (10^9^/L)	248.74 ± 78.67	242.89 ± 74.01	252.28 ± 81.20	1.961	0.050
HGB before biopsy (g/L)	127.11 ± 22.73	126.80 ± 22.89	127.30 ± 22.65	0.365	0.715
SCR before biopsy, μmol/L, median [IQR]	140.94 ± 128.13, 101.00 [73.00, 153.00]	136.76 ± 132.08, 96.00 [67.25, 146.00]	143.47 ± 125.70, 102.00 [76.00, 156.25]	0.859	0.391
ALB before biopsy (g/L)	33.80 ± 8.08	34.82 ± 7.51	33.18 ± 8.35	− 3.425	0.001
INR	0.97 ± 0.08	0.97 ± 0.07	0.96 ± 0.81	− 2.111	0.035
PT before biopsy (s)	11.39 ± 0.83	11.47 ± 0.84	11.34 ± 0.81	− 2.542	0.011
APTT before biopsy (s)	26.19 ± 3.30	26.67 ± 3.34	25.90 ± 3.25	− 3.852	< 0.001
eGFR [ml/min/1.73 m^2^]				− 0.685	0.493
< 15	399 (34.82)	155 (38.85)	244 (61.15)		
15 ~ 30	250 (21.81)	97 (38.80)	153 (61.20)		
31 ~ 59	308 (26.88)	109 (35.39)	199 (64.61)		
60 ~ 89	123 (10.73)	46 (37.40)	77 (62.60)		
≥ 90	66 (5.76)	25 (37.88)	41 (62.12)		
The number of biopsy needle pass (*n* = 853)				−1.162	0.245
1–2	43 (3.75)	24 (55.82)	19 (44.19)		
3–4	772 (67.36)	289 (37.44)	483 (62.56)		
≥ 5	38 (3.32)	17 (44.74)	21 (55.26)		
Diagnosis					
IgAN	296 (25.82)	136 (45.95)	160 (54.05)	11.564	0.001
MN	278 (24.26)	89 (32.01)	189 (67.99)	5.045	0.025
LN	87 (7.59)	45 (51.72)	42 (48.28)	7.888	0.005
HSPN	50 (4.36)	22 (44.00)	28 (56.00)	0.885	0.347
DKD	49(4.28)	11 (22.45)	38 (77.55)	5.067	0.024
MCD	45 (3.93)	17 (37.78)	28 (62.22)	0.000	0.991
CGN	23 (2.07)	6 (26.09)	17 (73.91)	1.347	0.246
Hypertensive renal disease	23 (2.07)	11(47.83)	12 (52.17)	1.025	0.311
Other diseases	313(27.31)	103(32.91)	210(67.09)	4.205	0.040
Postoperative urination				11.831	0.001
≤ 4 h	970 (84.64)	386 (39.79)	584 (62.21)		
> 4 h	176 (15.36)	46 (26.14)	130 (73.86)		
24-h intake and urine volume after puncture (ml)				−1.683	0.092
≤ − 500	414 (36.13)	167 (40.34)	247 (59.66)		
− 499 to 499	590 (51.48)	219 (37.12)	371 (62.88)		
≥ 500	142 (12.39)	46 (32.39)	96 (67.61)		

*BMI* body mass index, *SBP* systolic blood pressure, *DBP* diastolic blood pressure, *PLT* platelet, *HGB* hemoglobin, *SCR* serum creatinine, *ALB* Albumin, *INR* international normalized ratio, *PT* prothrombin time, *APTT* activated partial thromboplastin time, *eGFR* estimated glomerular filtration rate, *IgAN* immunoglobulin A nephropathy, *MN* membranous nephropathy, *LN* lupus nephritis, *RPGN* rapidly progressive glomerulonephritis, *HSPN* Henoch–Schönlein purpura nephritis, *DKD* diabetic kidney disease, *MCD* minimal change disease

The overall rate of post-biopsy hemorrhage was 37.70% (432/1146). Of those bleedings, minor bleeding after PRB was found in 337 (29.41%), middle bleeding 84 (7.33%), major bleeding 11(0.96%). Besides that, 96 patients (8.38%) reported their discomfort symptoms. There was no death. Statistics for post-biopsy complications are summarized in Table 3.

**Table 3:** Ultrasound-guided PRB complications and their incidence (*n* = 1146)

Complications	Occurrence cases	Incidence rate (%)
Post-biopsy bleedings	432	37.70
Minor bleeding	337	29.41
Middle bleeding	84	7.33
Major bleeding	11	0.96
Other discomfort symptoms	96	8.38

Note: other discomfort symptoms included nausea, vomiting, and abdominal pain

In order to correct confounding factors, we included variables with statistical differences in bivariate analysis into multivariable logistic regression analysis. Table 4 presents the multivariable logistic regression analysis results. It was observed that females were at significantly increased risk of hemorrhagic complication than males (OR = 2.017, CI = 1.531–2.658), while the risk for hemorrhagic complication significantly decreased as BMI and platelet before renal biopsy increased (OR = 0.956, CI = 0.924–0.989; OR = 0.998, CI = 0.996–1.000). As the APTT time prolonged, the risk for hemorrhagic complication significantly increased (OR = 1.072, CI = 1.023–1.123). Those patients whose albumin were higher also had higher risk for hemorrhagic complication than other patients (OR = 1.020, CI = 1.000–1.041). Specifically, postoperative urination within 4 h increased the risk for hemorrhagic complication (OR = 1.741, CI = 1.176–2.576).

**Table 4:** Multivariable logistic regression for hemorrhagic complication

Variables	β	SE	Wald	*P* value	OR [95% CI]
Gender					
Male	Reference	Reference	Reference	Reference	Reference
Female	0.702	0.141	24.840	< 0.001	2.017 [1.531, 2.658]
BMI (kg/m^2^)	− 0.045	0.017	6.633	0.010	0.956 [0.924, 0.989]
PLT before biopsy (10^9^/L)	−0.002	0.001	3.917	0.048	0.998 [0.996, 1.000]
ALB before biopsy (g/L)	0.020	0.010	3.916	0.048	1.020 [1.000, 1.041]
APTT before biopsy (s)	0.070	0.024	8.544	0.003	1.072 [1.023, 1.123]
Postoperative urination ≤ 4 h					
No	Reference	Reference	Reference	Reference	Reference
Yes	0.554	0.200	7.685		1.741 [1.176, 2.576]

## Discussion

For most patients, ultrasound-guided PRB is the preferred approach because it is less invasive compared with other approaches, while it can provide reliable gold standard for diagnosis, prognosis and management of renal disease. However, post-biopsy bleeding remains to be one of the most serious and common complications after PRB. Our study identified the overall rate of post-biopsy hemorrhage was 37.70%, which was higher than most previous studies [9–12] and almost similar as our team reported 14 years ago [13]. On the one hand, the potential reasons for higher incidence than other studies may include variance of definitions and methods for defining and assessing bleeding complications across studies [14, 15]. We performed a routine screening ultrasound for the patients’ safety on the third day after their PRB, which helped us find more post-biopsy asymptomatic hematoma and delayed bleeding complications. On the other hand, although the intervention was not required for minor bleeding, the rate of post-biopsy bleeding was still high, which indicated us needed to take more efforts to reduce the rate. Therefore, our team derived an appropriate preoperative assessment list based on our findings. Tailored careful observation after PRB was also set as a precaution against post-biopsy bleeding complications.

Prior studies have demonstrated that the common risk factors for renal biopsy complications focus on hypertension, coagulation status, high serum creatinine, age, gender, worsening of renal function and so on. In addition, some factors are still under debate, such as blood pressure, number of passes, and PT before biopsy. No difference was observed in this study between bleeding with age, hemoglobin concentration, kidney size, severity of kidney dysfunction, the type of kidney, comorbidities, or number of passes with the biopsy needle. Nevertheless, it was worth mentioning that although the research results showed an increased risk of bleeding as renal function decreases, the necessity of performing renal biopsy in patients with chronic kidney disease in stages 4–5 must be carefully determined based on benefits and potential risks. We deduced that the following factors of gender, BMI, platelet count before biopsy, albumin before biopsy, APTT before biopsy, and postoperative urination within 4 h might relate to post-biopsy bleeding complications. We found a significant association between gender and bleedings in our study, which was similar with recent studies [11, 14–16]. We also proved this association in our earlier study [13]. Female patients are more likely to bleed than male patients, which may be due to the fact that women have more loose tissue around the kidneys. In addition, it is possible that women’s kidneys are smaller than men’s, which might increase the chance of inadvertent injury to deeper structures during PRB [17].

Greater bleeding risk was observed in patients with higher APTT (OR: 1.072, 95% CI: 1.023, 1.123), higher serum albumin levels (OR: 1.020, 95% CI: 1.000, 1.041), while lower PLT (OR: 0.998, 95% CI: 0.996, 1.000) and lower BMI (OR: 0.956, 95% CI: 0.924, 0.989). In general, patients with prolonged activated partial thromboplastin time were more likely to have a bleeding complication. Lower PLT impaired effective hemostasis and delayed formation of a platelet plug at the site of renal biopsy. Fortunately, both of them could be modified by effective pre-biopsy management, such as using drugs, and active medical management. Therefore, nephrologists should recognize the potential risk of bleeding and other complications associated with the renal biopsy, and conduct the preventive measures to patients with prolonged activated partial thromboplastin time and (or) low PLT to avoid post-biopsy complications. Other pre-biopsy laboratory tests, such as SCR, HGB, and INR, seemed like no statistical significance in our study, but these indicators were significant for clinical practice, because these results could provide us essential information for evaluating the risk of complication and help us to take preventive measures to minimize the risk. Therefore, most of these guidelines make recommendations that routine care prior to biopsy involves measuring HGB, platelet count, INR and APTT. The study of Peters et al. [12] showed patients with lower BMI were at higher risk of major complications. A possible explanation for this might be that patients with higher BMI are less mobile. In addition, their adipose tissue may compress the wounded capsule of the kidney and the channel of the injection [12].

There is little published data on the clinical value of postoperative urination as we know. Interestingly, we found that postoperative urination within 4 h might increase the risk of post-biopsy bleeding complication (OR = 1.741, 95% CI: 1.176, 2.576). Although clinical practice varies at different centers, the patients were guided to drink more water than usual to decrease the risk of blood clot retention after PRB in our hospital. However, this turned out it might increase the risk of bleeding after PRB. Generally speaking, increased water intake leads to increased renal blood flow, which can flush out the clot after puncture, and reduce the risk of urinary tract obstruction. Renal blood flow might be not conducive to coagulation at the renal puncture channel. Another possible explanation for this result is that body movement increased the risk of bleeding. Despite being well trained how to urinate in bed, the patients had to move their bodies when they needed to urinate, which increased the risk of bleeding. The sooner the patient urinated, the earlier body movement occurred, and therefore, the risk of bleeding increased. This finding may support the clinical practice that the patient should be supine for 4 to 6 h and then remain at bedrest overnight.

## Conclusions

In summary, ultrasound-percutaneous renal biopsy contributes to identify potential targets for therapy. Our analysis finds that the incidence of post-biopsy bleeding complication is 37.70%, and its risk is associated with female, lower BMI, prolonged APTT, higher albumin, lower platelet and postoperative urination within 4 h. The findings highlighted the importance of perioperative management for renal biopsy, including adequate risk assessment, tailored careful observation after PRB. In addition, we suggested that minimum set of pre-biopsy laboratory tests for bleeding tendency should include platelet count, APTT, and albumin. Besides that, the patient should be supine for 4 to 6 h and then remain at bedrest overnight while nurse conducting 24-h diet records (including water), and urinary volume observation to keep fluid balance. In spite of these limitations, this study provides useful information on the incidence and risk factors of post-biopsy bleeding complications from a real-world dataset in one of the largest biopsy series in China.

## Limitations of study

Some limitations of the study deserve mentioning. First, it was a single-center study. Second, this study was a retrospective study, which would lead to bias and limited data. Importantly, we could not analyze certain covariates of interest, such as timing of bleeding and patients’ adherence. However, in the process of collecting data, we try our best to ensure the accuracy of pathological results and the integrity of database data, so as to avoid case selection bias and result measurement bias as much as possible.

## Data Availability

The data that support the findings of this study are available on reasonable request from the corresponding author.

## Abbreviations

AKI: Acute kidney injury
ALB: Albumin
APTT: Activated partial thromboplastin time
BMI: Body mass index
CKD: Chronic kidney disease
DBP: Diastolic blood pressure
DKD: Diabetic kidney disease
eGFR: Estimated glomerular filtration rate
HGB: Hemoglobin
HSPN: Henoch–Schönlein purpura nephritis
HTN: Hypertensive renal disease
IgAN: Immunoglobulin A nephropathy
INR: International normalized ratio
LN: Lupus nephritis
MCD: Minimal change disease
MN: Membranous nephropathy
PLT: Platelet
PRB: Percutaneous renal biopsy
PT: Prothrombin time
RPGN: Rapidly progressive glomerulonephritis
SBP: Systolic blood pressure
SCR: Serum creatinine
